# Key Roles in the Process of Extravasation and Colonization: Acyl‐coenzyme A Synthetase Long‐chain Family Member 4 and Polyunsaturated Lipids

**DOI:** 10.1002/mco2.70264

**Published:** 2025-06-15

**Authors:** Jiayu Han, Jie Zhang, Yicheng Chen

**Affiliations:** ^1^ Department of Urology Sir Run Run Shaw Hospital, School of Medicine, Zhejiang University Hangzhou China

1

In a recent study published in *Cell*, Yilong Zou et al. [1] revealed dual functions of polyunsaturated fatty acyl (PUFA)‐lipid in metastatic cancer cells. Acyl‐coenzyme A (CoA) synthetase long‐chain family member 4 (ACSL4), a PUFA‐lipid biosynthesis enzyme, promotes hematogenous metastasis by enhancing membrane fluidity and cell invasiveness. Concurrently, elevated levels of PUFA‐lipid induce reliance of cancer cells on enoyl‐CoA delta isomerase 1 (ECI1) and enoyl‐CoA hydratase 1 (ECH1), enzymes preparing UFAs for β‐oxidation. Dual inhibition of ACSL4/ECH1 effectively suppresses tumor metastasis.

The majority of cancer patients succumb to metastatic disease rather than primary tumor burden [2]. Moreover, metastatic cancers remain largely incurable in clinical settings, ultimately leading to death due to systemic organ failure [2]. Extravasation, dormancy, and colonization are three major steps of metastasis [2] alongside other critical processes such as epithelial‐to‐mesenchymal transition. Factors impacting extravasation include increased cell motility and altered lipid phase behavior. At the same juncture, the metabolism of fatty acid and acyl‐CoA plays an extremely significant role in the progression of cancer cell growth and metastasis [3].

Yilong Zou et al. conducted an analysis utilizing the Cancer Metastasis Map and the Cancer Therapeutics Response Portal to find whether metastatic cancer cells are more sensitive to specific cytotoxic compounds within the cancer cell lines listed in the dataset. These well‐established databases serve as invaluable resources for oncology research, offering comprehensive datasets and insights that are worthy of further exploration. The results demonstrated that ovarian cancer cells exhibiting higher metastatic potential displayed increased sensitivity to ferroptosis induction. Subsequently, they utilized clinically collected metastatic samples to validate that ovarian cancer cells with higher metastatic potential showed increased susceptibility to ferroptosis and elevated levels of unsaturated lipids. Notably, emerging evidence indicates that oncogenic driver mutations may simultaneously prime cancer cells for ferroptosis vulnerability in breast and gastric adenocarcinomas [4].

To investigate the function of PUFA‐lipids and ferroptosis‐sensitizing factors in metastatic progression, an ovarian cancer metastasis model was established through intraperitoneal injection of GFP‐firefly‐luciferase‐labeled ES‐2 cells, a clear‐cell carcinoma line derived from malignant ascites of ovarian cancer patients. Utilizing lipidomic analysis, the researchers found that PUFA‐lipids increased in pulmonary metastatic lesions, without a corresponding change in the abundance of lipid droplets. This suggested a potential relationship between ferroptosis susceptibility and increased PUFA‐lipids. To further minimize variability in the de novo model, GFP⁺ cells obtained through primary in vivo selection underwent secondary enrichment via intraperitoneal injection. Following the two in vivo selection cycles, the metastatic penetrance reached 100%. This methodology employs an in vivo selection strategy to isolate and propagate highly metastatic cell subpopulations, facilitating the investigation of tumor clones with enhanced metastatic potential.

Following the isolation of metastasis‐competent cellular subpopulations, Yilong Zou et al. conducted a CRISPR screen, which is a stable method for detecting specific genes involved in cancer evolution [5], to hierarchically characterize the core regulatory molecules that orchestrate metastatic cascade progression. They performed the first CRISPR screen using a sgRNA library targeting phosphatases to ensure a high representation of sgRNA changes between bulk tumors and metastatic lesions. To pinpoint metabolic vulnerabilities within metastatic ovarian cancers, the second CRISPR screen interrogating core metabolic genes was conducted. Another CRISPR screen, which was performed using a sgRNA library, a library encompassing 100 top‐depleted or ‐enriched genes from prior screens, revealed that four genes–nicotinamide nucleotide adenylyltransferase 1(NMNAT1), hydroxyacyl‐CoA dehydrogenase trifunctional multienzyme complex subunit b (HADHB), protein tyrosine phosphatase receptor type G (PTPRG) and ATPase H+ transporting V1 subunit H (ATP6V1H) played a role in membrane lipid metabolism and fatty acid oxidation in both lung and liver metastases. In lung‐metastasis‐specific regulators, ACSL4, which took part in fatty acid activation showed great potential for facilitating metastasis. CRISPR screening represents a paradigm‐shifting approach that has uniquely enabled the systematic discovery of novel genetic determinants and their mechanistic underpinnings, which have remained refractory to conventional experiments. To confirm ACSL4's functional role in the process of metastasis, the mRNA expression from a recent single‐cell RNA sequencing dataset was examined and then ACSL4‐depleted ovarian cancer cells were intravenously injected to find a significant reduction in the lung metastatic burden. All these experiments collectively validated the critical role of ACSL4 in promoting metastasis. To delineate the stage‐specific regulatory role of ACSL4, sgACSL4 cells were injected intravenously to model the later stages of metastasis, specifically extravasation and colonization. The results showed that mice engrafted with sgACSL4 ovarian cancer cells demonstrated smaller metastatic lesions and unusual intravascular localization. Compared with the luminescence signal after perfusion, mice implanted with sgACSL4 cells exhibited a dramatically weaker signal. The application of the ACSL4 inhibitor, PRGL493, in therapy underscores the crucial role of ACSL4 in promoting cell extravasation. Subsequently, the viscosity of cell membranes, a critical factor for cancer cell deformation during metastasis, was assessed in ACSL4 depletion cells. Then the parental cells were supplemented with arachidonic acid to increase PUFA‐lipid content, with enhanced metastatic extravasation being observed. All the experiments showed that ACSL4, together with the PUFA‐lipidome, enhanced metastatic extravasation via altering membrane viscosity and increasing cancer cell invasiveness. To broaden the findings, they repeated experiments in liver and breast cancer cell lines, consistently observing the same outcome.

Due to the high cancer burden, targeting ACSL4 alone proved insufficient for therapeutic efficacy. Considering the variable score of ACSL4 in the mini‐CRISPR screen, the researchers suspected that ACSL4 may work only at the initial stage of metastasis. To further investigate the late‐stage mechanisms of metastasis in high PUFA‐lipid cancer cells, candidate genes analysis revealed that ECH1 and ECI1 knockout could significantly shrink subcutaneous ovarian tumors. Leveraging the public gene expression datasets, the researchers confirmed the involvement of the UFA β‐oxidation pathway in cancer progression. In the TCGA dataset, the poor prognosis of ovarian cancer could be predicted by the combined high expression levels of abhydrolase‐domain‐containing 6, acylglycerol lipase (ABHD6), malate dehydrogenase 1(MDH1), ECI1, and ECH1 mRNAs. To validate the hypothesis that dual inhibition of ACSL4 and ECH1 could further restrict the metastasis of tumors, double‐knockout cells were constructed for intravenous injection. The results confirmed that dual inhibition of ACSL4 and ECH1 suppressed metastasis by blocking UFA esterification and β‐oxidation.

In summary, this study highlighted the key role of lipid metabolism, particularly PUFA in tumor metastasis. The research demonstrated that ACSL4 played a significant role in facilitating extravasation and invasiveness of cancer cells and PUFA‐enriched cancer cells relied on ECI1/ECH1‐mediated β‐oxidation for survival during metastatic colonization. Co‐inhibiting of ACSL4 and ECH1 may offer new treatment options for ovarian cancer and other cancer types (Figure 1). Therefore, addressing the unique metabolic needs of these cancer cells, particularly their reliance on ATP, could inhibit their survival and expansion within the metastatic environment.

**FIGURE 1:**
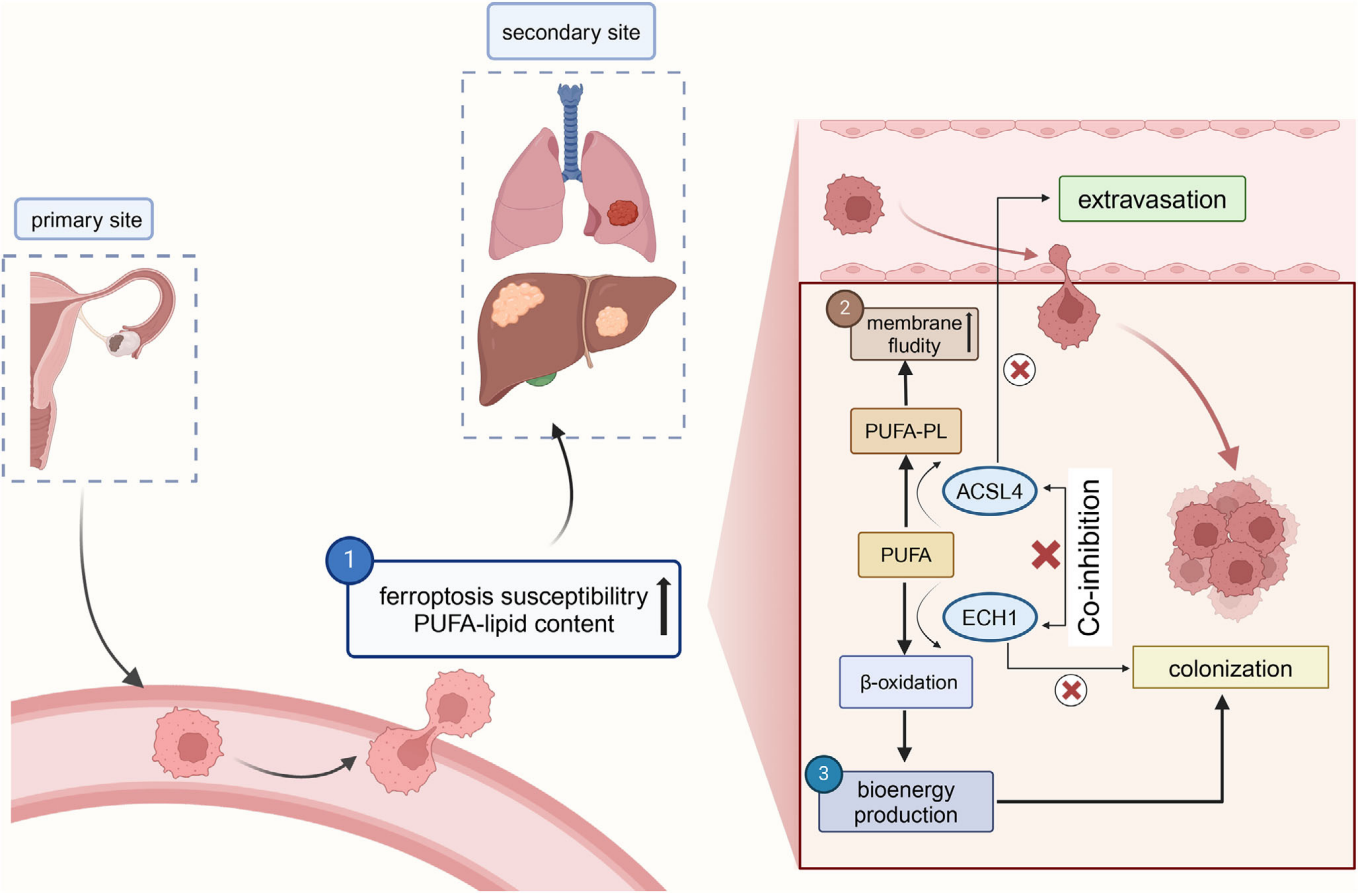
The mechanism of polyunsaturated fatty acyl (PUFA)’s dual role in cancer metastasis. The cells transferred from the ovary to the lung and liver showed higher ferroptosis susceptibility and PUFA‐lipid content. In the process of extravasation, acyl‐coenzyme A synthetase long‐chain family member 4 (ACSL4) will increase the membrane fluidity and invasiveness. In the process of colonization, enoyl‐CoA hydratase 1 (ECH1) will catalyze β‐oxidation to produce bioenergy for colonizing. Co‐inhibition of ACSL4 and ECH1 provides a new therapy for ovarian cancer.

However, the conclusions of this study are subject to several limitations. First, the tumor modeling was predominantly carried out in immunodeficient mice, and the tumorigenesis method bypassed the early stages of metastasis. Second, there were no direct pharmacological methods to specifically target the UFA β‐oxidation and ACSL4 pathways in vivo.

Nevertheless, this study unveiled profound mechanistic insights into the pivotal role of unsaturated lipid metabolic pathways in facilitating tumor metastasis. The findings not only elucidate molecular phenomena with exceptional clarity but also demonstrate its remarkable generalizability across diverse cancer types. This discovery advances understanding of metabolic regulation in metastasis and opens new avenues for targeted therapies.

## Author Contributions

**J.H**. and **J.Z**. conceptualized, wrote, and edited the manuscript. **J.H**. and **J.Z**. designed and created the figure using BioRender. **Y.C**. and **J.Z**. revised the manuscript. All authors have read and approved the final manuscript.

## Conflicts of Interest

The authors declare no conflicts of interest.

## Ethics Statement

The authors have nothing to report.

## Data Availability

The authors have nothing to report.
